# Fluorescence-based thermal shift data on multidrug regulator AcrR from *Salmonella entericasubsp. entrica serovar Typhimurium str. LT2*

**DOI:** 10.1016/j.dib.2016.03.003

**Published:** 2016-03-09

**Authors:** Babu A. Manjasetty, Andrei S. Halavaty, Chi-Hao Luan, Jerzy Osipiuk, Rory Mulligan, Keehwan Kwon, Wayne F. Anderson, Andrzej Joachimiak

**Affiliations:** a European Molecular Biology Laboratory (EMBL), Grenoble Outstation, 71 Avenue des Martyrs, F-38042 Grenoble, France; b Unit of Virus–Host Cell interactions (UVHCI), University of Grenoble Alpes, F-38042 Grenoble, France; c Biochemistry and Molecular Genetics, Feinberg School of Medicine, Northwestern University, 303 East Chicago Avenue, Chicago, IL 60611, United States; d Center for Structural Genomics of Infectious Diseases (CSGID), 303 East Chicago Avenue, Chicago, IL 60626, United States; e High Throughput Analysis Laboratory, Northwestern University, 2205 Tech Drive, Evanston, IL 60208, United States; f Computational Institute, The University of Chicago, 5735 South Ellis Avenue, Chicago, IL 60637, United States; g Structural Biology Center, Argonne National Laboratory, 9700 South Cass Avenue, Argonne, IL 60439, United States; h Infectious Diseases, J. Craig Venter Institute, Rockville, MD, United States

**Keywords:** Fluorescence-based thermal shift analysis, Infectious diseases, Transcriptional regulator, TetR, AcrR, *Salmonella enterica*, High-throughout screening

## Abstract

The fluorescence-based thermal shift (FTS) data presented here include Table S1 and Fig. S1, and are supplemental to our original research article describing detailed structural, FTS, and fluorescence polarization analyses of the *Salmonella entericasubsp. entrica serovar Typhimurium str. LT2* multidrug transcriptional regulator AcrR (StAcrR) (doi:10.1016/j.jsb.2016.01.008) (Manjasetty et al., 2015 [1]). Table S1 contains chemical formulas, a Chemical Abstracts Service (CAS) Registry Number (CAS no.), FTS rank (a ligand with the highest rank) has the largest difference in the melting temperature (Δ*T*_m_), and uses as drug molecules against various pathological conditions of sixteen small-molecule ligands that increase thermal stability of StAcrR. Thermal stability of human enolase 1, a negative control protein, was not affected in the presence of various concentrations of the top six StAcrR binders (Fig. S1).

**Specifications Table**

**Table:** 

Subject area	*Chemistry, Biology*
More specific subject area	*Protein-ligand interactions*
Type of data	*Tables, figure*
How data was acquired	*Fluorescence-based thermal shift (FTS) analysis. 384-well PCR plates; an Echo550 acoustic transfer robot (Labcyte, Sunnyvale, CA); real-time PCR machine CFX384 (Bio-Rad Laboratories, Hercules, CA); in-house ExcelFTS software.*
Data format	*Analyzed*
Experimental factors	*High concentration protein stocks were diluted in the FTS analysis buffer*
Experimental features	*The FTS analysis was performed in high-throughput mode using semi-automatic approach*
Data source location	*Chicago*
Data accessibility	*Data is within this article.*

## Value of the data

• FTS data present potential StAcrR small-molecule binders that may affect the protein׳s structure and function.
• Researchers in the field may utilize employed high-throughput FTS approach in screening small-molecule binders for homologous multidrug regulators from the TetR family.
• Collectively, presented data could be exploited for the design of the StAcrR inhibitors.

## 1 Data

Here, we present FTS data featuring multidrug-binding nature of the transcriptional regulator StAcrR. The top sixteen binders of StAcrR, as shown in Table S1, were identified by the FTS analysis from a library of 320 unique ligands. Human enolase 1, a negative control protein (Fig. S1), did not bind the top six binders of StAcrR, while implying their specific interactions with StAcrR. The remaining compounds were not tested against human enolase 1. Theoretical data for Table S2 were obtained from National Center for Biotechnology Information PubChem Compound Database (https://pubchem.ncbi.nlm.nih.gov/compound/) and include chemical properties such as topological polar surface (Å^3^), number of hydrogen bond donors/acceptors, XLogP3, and molecular weight (g/mol) of the top six StAcrR FTS hits, dequalinium chloride, proflavine, ethidium bromide and rhodamine 6G. The latter three molecules were identified as the StAcrR binders employing a fluorescence polarization experimental approach (see our original publication [1]).

## 2 Experimental design, materials and methods

The FTS assay was run in 384-well PCR plates using an Echo550 acoustic transfer robot (Labcyte, Sunnyvale, CA) for dispensing a dimethyl sulfoxide stock of ligands to assay plates that contain 10 μl of a mixture of StAcrR (1 μg) and 2.5×SYPRO Orange fluorescence dye (Invitrogen, Carlsbad, CA) in 100 mM HEPES buffer pH 7.5 and 150 mM NaCl. Thermal scanning (from 10 to 80 °C at 1.5 °C min^−1^ ramp rate) on a real-time PCR machine CFX384 (Bio-Rad Laboratories, Hercules, CA) was coupled with fluorescence detection every 10 s.

A test molecule, dequalinium, bound at 40 μM that prompted us to screen a 320-molecule subset of the Spectrum library (Micro Source Discovery, Gaylordsville, CT, hereafter referred as SPC2-ECH008), dequalinium belongs to. The best binders were selected based on Δ*T*_m_, reduction of the background reading, and shape of the melting curve. The top six hits were further subjected to a dose-dependent response analysis using their 2.5, 5, 10, 25, 50, 75 and 100 μM concentrations. Human enolase 1 (1.2 μg in 10 μl assay mixture) was used as a negative control protein and tested against the top six binders at 10, 25 and 50 100 μM concentrations. FTS data were analyzed with the in-house ExcelFTS software.
