# Supplementary material for: Fluorescence-based thermal shift data on multidrug regulator AcrR from Salmonella entericasubsp. entrica serovar Typhimurium str. LT2
Source: Data Brief. 2016 Mar 9;7:537–9. doi: 10.1016/j.dib.2016.03.003 (PMC4796704; doi:10.1016/j.dib.2016.03.003)
Supplement: Supplementary file 2 — Supplementary material [file mmc2.pdf]

Table S1

Small-molecule binders of StAcrR as assayed by FTS.

Rank	***Molecule (CAS No.)***	***Structure***	***ΔT_m (℃)***	***Uses***
[a]Negative control	*Diflubenzuron (35367-38-5)*	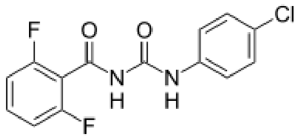	*N/A*	*Benzoylurea-type insecticide of the benzamide class.*
1	*Fulvestrant (129453-61-8)*	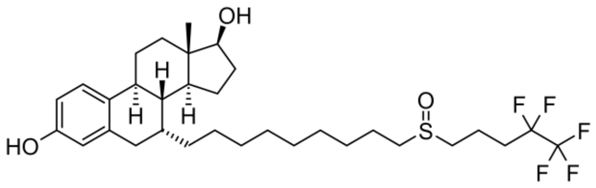	*6.23*	*Treatment of hormone receptor-positive metastatic breast cancer in postmenopausal women.*
2	*Idebenone (58186-27-9)*	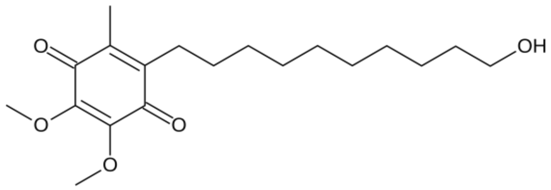	*5.73*	*Treatment of Alzheimer's disease and other cognitive defects. Early clinical studies completed for Friedreich's ataxia and Duchenne muscular dystrophy.*
3	*Triclabendazole (68786-66-3)*	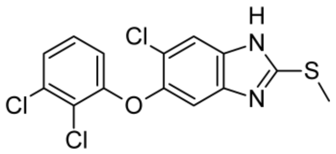	*5.58*	*Displays high efficacy against both immature and adult liver flukes.*

4	*Ritonavir (155213-67-5)*	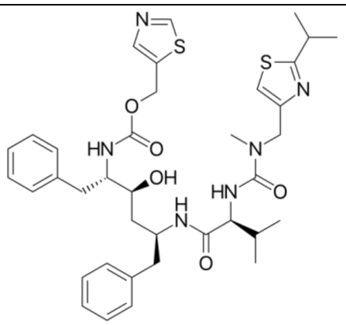	*5.23*	*An antiretroviral medication to treat HIV/AIDS*
5	*Estramustine (2998-57-4)*	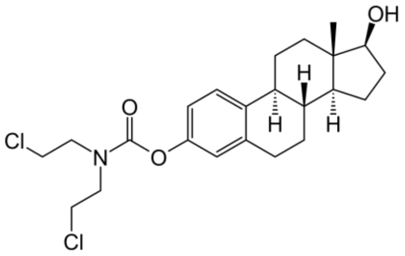	*4.14*	*An antimicrotubule chemotherapy agent used to treat prostate cancer*
6	*Prasterone (53-43-0)*	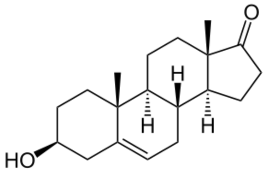	*4.09*	*The most abundant circulating steroid hormone in humans A metabolic intermediate in the biosynthesis of the androgen and estrogen sex steroids.*
7	*Vilazodone hydrochloride (163521-12-8)*	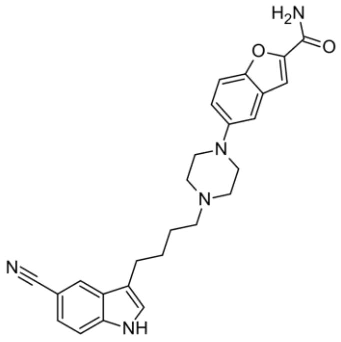	*3.71*	*A serotonergic antidepressant (treatment of major depressive disorder)*
8	*Quinestrol (152-43-2)*	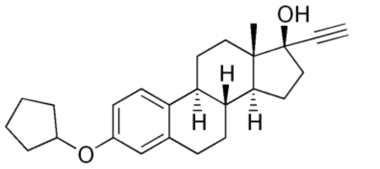	*2.72*	*Hormone replacement therapy, and occasionally to treat breast cancer and prostate cancer*

9	*Mebhydrolin (524-81-2) naphthalene sulfonate (532-02-5)*	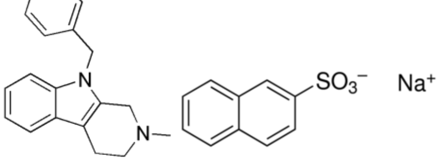	*2.55*	*Mebhydrolin or mebhydroline is an antihistamine*
10	*Finasteride (98319-26-7)*	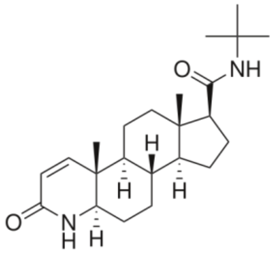	*2.06*	*Benign prostatic hyperplasia and male pattern baldness*
11	*Pentamidine isethionate salt (140-64-7)*	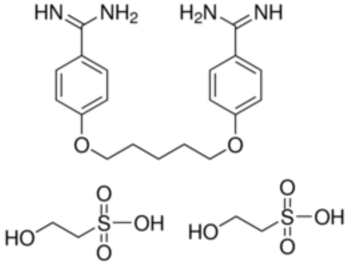	*2.04*	*Treatment of Pneumocystis pneumonia. Stage I infection with Trypanosoma brucei gambiense*
12	*Docusate sodium (577-11-7)*	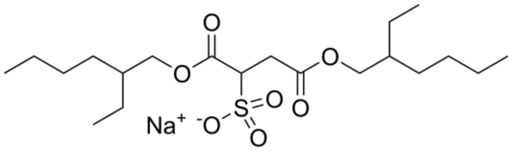	*1.85*	*A laxative used to treat constipation*
13	*Cyproterone (2098-66-0)*	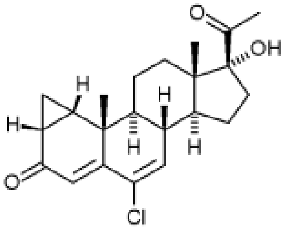	*1.71*	*An acylated derivative, cyproterone acetate, is widely used clinically as an antiandrogen and progestin*
14	*Dequalinium chloride (522-51-0)*	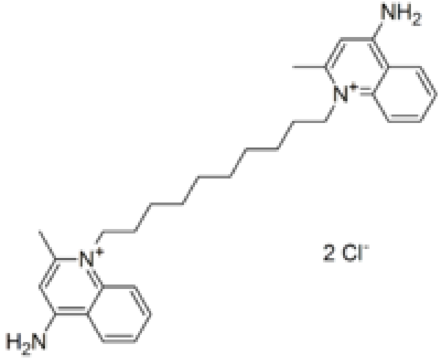	*1.59*	*Active ingredient of Dequadin (antiseptic, disinfectant, topical bacteriostat, antifungal) and Fluomizin (treatment of bacterial*

				vaginosis)
14	*Tepoxalin (103475-41-8)*	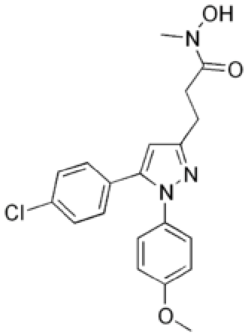	*1.59*	*Nonsteroidal anti-inflammatory drug (veterinary use)*
16	*Ziprasidone mesylate (146939-27-7)*	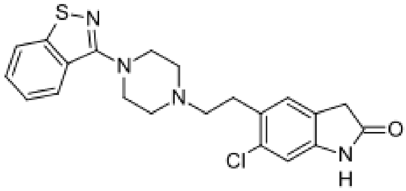	*1.56*	*Atypical antipsychotic (treatment of schizophrenia, acute mania and mixed states associated with bipolar disorder)*

[a]*Diflubenzuron did not shift T_m of StAcrR.*

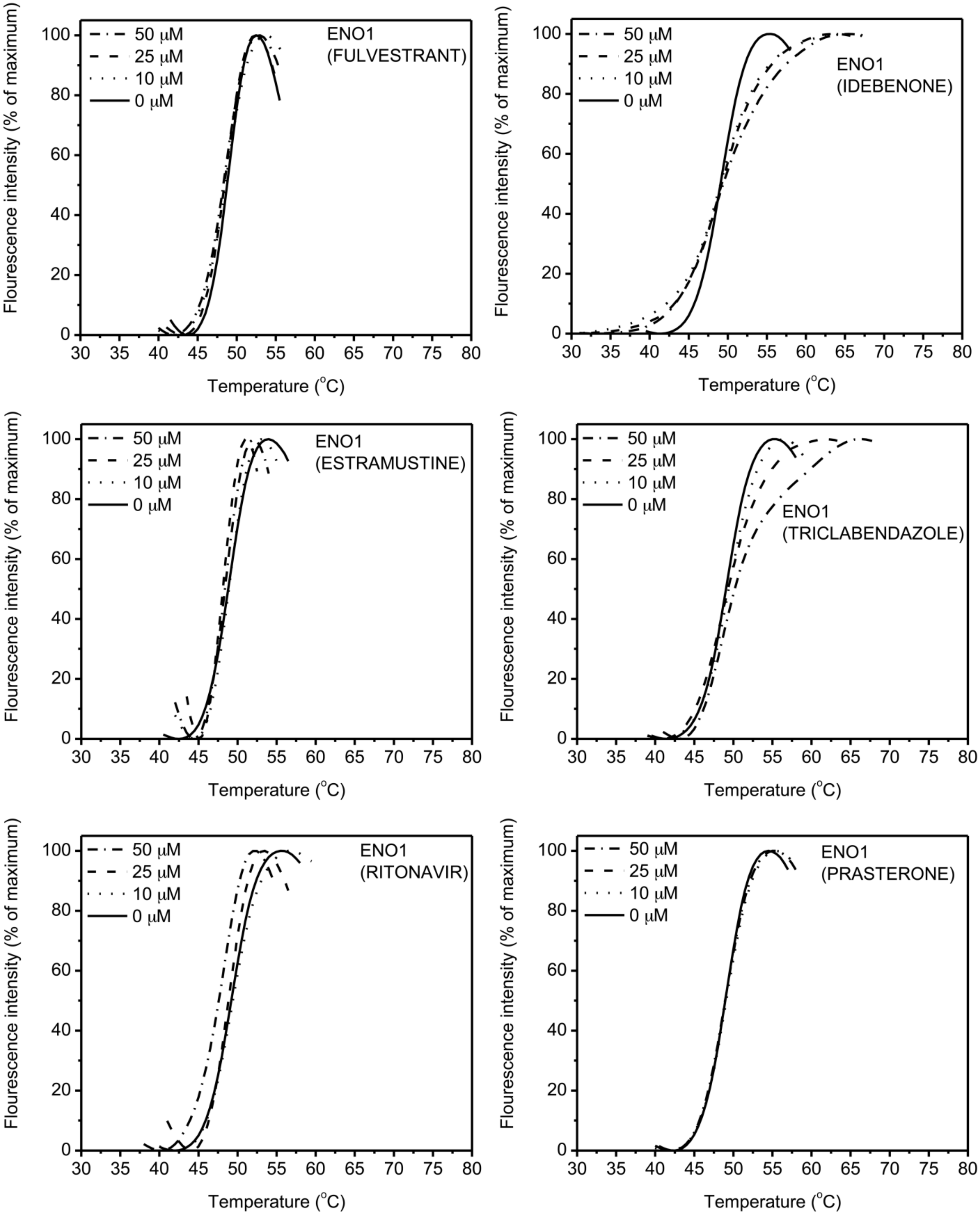
ENO1
(FULVESTRANT)
ENO1
(IDEBENONE)
ENO1
(ESTRAMUSTINE)
ENO1
(TRICLABENDAZOLE)
ENO1
(RITONAVIR)
ENO1
(PRASTERONE)
50 μM
25 μM
10 μM
0 μM
Flourescence intensity (% of maximum)
Temperature (°C)
0
20
40
60
80
100
30
35
40
45
50
55
60
65
70
75
80

Fig. S1. *Dose-dependent FTS melting curves of a negative control protein, human enolase 1 (ENO1), in the presence of 10, 25 and 50 μM concentrations of the top six StAcrR binders identified by the FTS approach.*

Table S2

Chemical properties of the top six FTS hits, dequalinium, Pf, Et and R6G.

Data obtained from National Center for Biotechnology Information. PubChem Compound Database (https://pubchem.ncbi.nlm.nih.gov/compound/)

Ligand	***Topological polar surface (Å^2)***	***No. of hydrogen bond donors***	***No. of hydrogen bond acceptors***	***XLogP3[a]***	***Molecular weight (g/mol)***
Fulvestrant	*77*	*2*	*9*	*9.2[b]*	*606.7*
Idebenone	*73*	*1*	*5*	*4.3*	*338.4*
Triclabendazole	*63*	*1*	*3*	*5.7*	*359.6*
Ritonavir	*202*	*4*	*9*	*6*	*720.9*
Estramustine	*50*	*1*	*3*	*4*	*440.4*
Prasterone	*37*	*1*	*2*	*3.2*	*288.4*
Pf	*65*	*2*	*3*	*1.8*	*209.2*
Et	*56*	*2*	*2*	*4.2*	*314.4*
R6G	*60*	*2*	*5*	*N/A*	*479.0*
Dequalinium chloride	*60*	*2*	*4*	*N/A*	*527.6*

[a]XLogP3 is a method for the logarithm of the ratio of the concentrations of the un-ionized solute in the solvents or a measure of lipophilicity (LogP) calculation [2]. [b]Values in red are violations of the Lipinski's rule of five [3].

References

[2] T. Cheng, Y. Zhao, X. Li, F. Lin, Y. Xu, X. Zhang, Y. Li, R. Wang, L. Lai, Computation of octanol–water partition coefficients by guiding an additive model with knowledge, J. Chem. Inf. Model. 47 (2007) 2140–2148.

[3] C.A. Lipinski, F. Lombardo, B.W. Dominy, P.J. Feeney, Experimental and computational approaches to estimate solubility and permeability in drug discovery and development settings, Adv. Drug. Deliv. Rev. 23 (2001) 3–25.
